# Mediating role of circulating inflammatory proteins in the effect of immune cells on esophageal cancer risk: A Mendelian randomization study

**DOI:** 10.1097/MD.0000000000040374

**Published:** 2024-11-01

**Authors:** Jinzhou Guo, Gao Si, Xuejie Song, Fuchun Si

**Affiliations:** a Henan University of Chinese Medicine, Zhengzhou, Henan, China; b Laboratory of TCM Syndrome and Prescription Signaling, Academy of Zhongjing, Zhengzhou, Henan, China; c Henan Key Laboratory of TCM Syndrome and Prescription Signaling, Henan International Joint, Zhengzhou, Henan, China; d Department of Orthopedic, Peking University Third Hospital, Beijing, China.

**Keywords:** BWMR analysis, causal inference, circulating inflammatory proteins, esophageal cancer, immune cells, mediation analysis, MR analysis

## Abstract

The immune system and inflammatory processes play crucial roles in the development of esophageal cancer (EC). This study aimed to investigate the causal relationships between 731 immune cell phenotypes, 91 circulating inflammatory proteins, and EC, with a particular focus on the mediating role of circulating inflammatory proteins. Utilizing public genetic data, we applied a 2-sample Mendelian Randomization (MR) method to examine the causal relationships between 731 immune cell phenotypes, 91 circulating inflammatory proteins, and EC. Comprehensive sensitivity analyses were conducted to assess the robustness, heterogeneity, and horizontal pleiotropy of the MR results. Additionally, a 2-step MR method was employed to quantify the impact and proportion of immune cell phenotypes mediated by circulating inflammatory proteins on EC. Eleven immune cell phenotypes and 1 inflammatory cytokine were found to have causal relationships with EC, with results stable across all sensitivity analyses. Mediation analyses revealed that only 2 cell phenotypes had causal relationships with EC through interleukin-10: CD3 on human leukocyte antigen-DR (HLA-DR)+ T cells (mediation effect = −0.009; mediation proportion = 12.01%) and monocytic myeloid-derived suppressor cell absolute count (mediation effect = 0.018; mediation proportion = 18.97%). This study enhances the understanding of the causal relationships between immune cells, circulating inflammatory proteins, and EC. The findings highlight the potential mediating role of interleukin-10, providing new insights into the mechanisms by which immune cells may influence esophageal tumorigenesis.

## 1. Introduction

Esophageal cancer (EC) is a common malignant tumor of the digestive system. According to GLOBOCAN 2020, EC ranks seventh globally in incidence and sixth in mortality, with the highest rates in East Asia.^[1]^ EC is known for its aggressive nature and poor prognosis. The overall 5-year survival rate is less than 30%, dropping to 5% for advanced cases.^[2]^ Survival rates vary significantly depending on the stage of diagnosis and availability of medical resources. EC shows notable geographical variation, with high-incidence regions known as the “esophageal cancer belt.” There are 2 primary belts: one in Central and East Asia, and the other in Eastern and Southern Africa.^[3]^ Asia, especially East Asia, faces the highest risk of EC due to a complex interplay of dietary habits, environmental exposures, and genetic factors.^[4,5]^ The immune system and inflammatory processes also play significant roles in cancer development.^[6]^ Understanding the relationships between immune cells, circulating inflammatory proteins, and EC is crucial for elucidating the mechanisms of carcinogenesis and developing targeted therapies.

Chronic inflammation contributes to EC initiation and progression by causing cellular and DNA damage.^[7,8]^ Immune modulation is essential in EC, with immune cells and factors playing key roles in tumor control and metastasis.^[9,10]^ T cells, nn cells, and B cells are involved in tumor response. T cells, including cytotoxic T lymphocytes and Tregs, recognize and destroy cancer cells.^[11,12]^ NK cells target and lyse malignant cells without prior sensitization.^[13]^ B cells have both anti-tumor and pro-tumor functions.^[14,15]^ Our previous Mendelian study found a causal relationship between immune cells and EC risk, but the analysis was limited to an Asian cohort, restricting generalizability. Inflammatory proteins are key mediators of immune cell function.^[16]^ Immune cells secrete and regulate these proteins to exert biological effects.^[17,18]^ Circulating inflammatory proteins are crucial in EC occurrence, development, and treatment. They modulate immune cell activity and influence the tumor microenvironment, participating in EC initiation, progression, and therapeutic responses.

Mendelian Randomization (MR) is a statistical method using single-nucleotide polymorphisms (SNPs) as instrumental variables (IVs) to infer causal relationships between exposure risk factors and outcomes.^[19]^ MR uses genetic variants determined at conception to infer causal effects of modifiable risk factors, reducing confounding and establishing credible associations.^[20]^ To enhance causal inference, Bayesian-Weighted Mendelian Randomization (BWMR) was applied.^[21]^ BWMR accounts for uncertainties in weak polygenic effects and addresses IV assumption violations through Bayesian-weighted outlier detection.^[21,22]^ This study aimed to explore causal relationships between immune cells, circulating inflammatory proteins, and EC, focusing on mediating effects. A 2-sample MR analysis was performed to estimate the causal effects of immune cell signatures and circulating inflammatory proteins on EC. A 2-step MR analysis identified circulating inflammatory proteins as potential mediators between immunophenotypes and EC. This study examined how immune cell phenotypes influence EC, deepening our understanding of the causal impact of peripheral immunity on EC risk. The findings offer new insights into therapeutic strategies and preventive measures.

## 2. Methods

### 2.1. Study design

**Figure 1.**
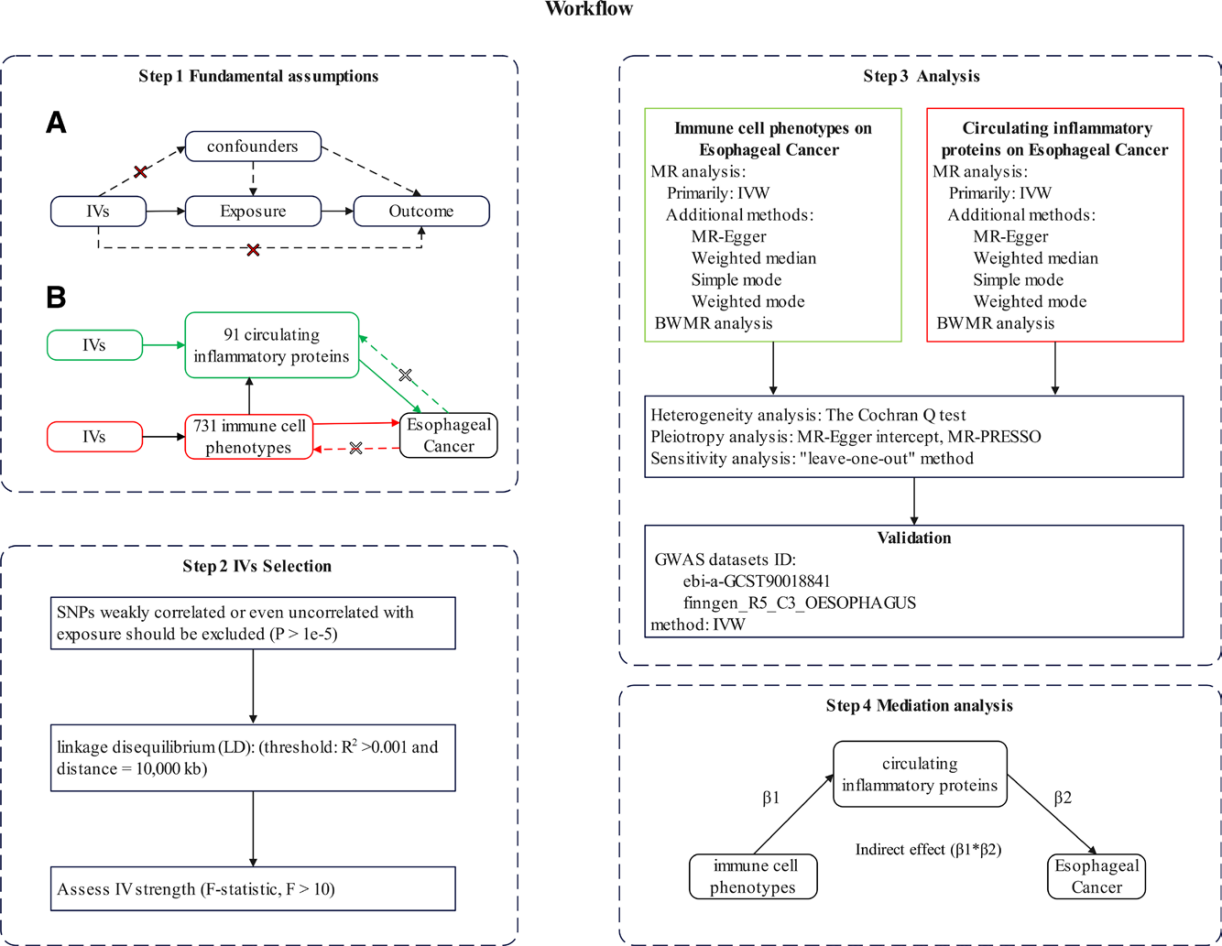
The steps of MR analysis. BWMR = Bayesian-Weighted Mendelian Randomization, IVW = inverse variance weighting, MR = Mendelian randomization, MR-PRESSO = Mendelian Randomization Pleiotropy RESidual Sum and Outlier, SNP = single-nucleotide polymorphism.

This study used a 2-sample MR approach to explore causal relationships between 731 immune cell phenotypes, 91 circulating inflammatory proteins, and EC. A 2-step MR approach identified the mediation effect of circulating inflammatory proteins on the relationship between immune cell phenotypes and EC. To ensure robust causal inference, IVs had to meet 3 assumptions: Each IV must be directly associated with the exposure factors; IVs should not correlate with confounders between exposure and outcome; IVs should not affect the outcome through pathways other than the exposure.^[23]^ All MR analyses used publicly available summary statistics, eliminating the need for additional ethical approval or informed consent. Figure 1 illustrates the analytical principles and procedures.

## 3. Data sources

### 3.1. Exposure data sources

Genome-Wide Association Study (GWAS) summary statistics for immune traits were retrieved from the GWAS Catalog (accession numbers GCST90001391–GCST90002121).^[24]^ The original GWAS for immune traits used data from 3757 European individuals, ensuring no cohort overlap. These datasets encompass 731 immunophenotypes, including B cells, dendritic cells (DCs), T-cell maturation stages, monocytes, myeloid cells, Treg panels, and T cells, B cells, and natural killer cells. GWAS data for 91 circulating inflammatory proteins were obtained from a meta-analysis of 14,824 participants of European ancestry (accession numbers GCST90274758–GCST90274848).^[25]^ More details are provided in S1 Table, Supplemental Digital Content, http://links.lww.com/MD/N852, and S2 Table, Supplemental Digital Content, http://links.lww.com/MD/N853.

## 4. Outcome data sources

GWAS summary statistics for EC were obtained from 3 cohorts. Two cohorts were sourced from the OpenGWAS database (https://gwas.mrcieu.ac.uk/): an Asian cohort for the initial analysis and a European cohort for validation (accession numbers ebi-a-GCST90018621 and ebi-a-GCST90018841).^[26]^ The third cohort was obtained from the FinnGen database (https://finngen.fi/), including genotypic data from 500,000 Finnish individuals. Positive results from the Asian cohort were validated in the European cohort and the FinnGen database to enhance generalizability. Details of the GWAS datasets are provided in Table 1.

**Table 1:** The summary of the GWAS dataset for the EC.

GWAS ID	Consortium	Participants	Cases	Controls	Population
ebi-a-GCST90018621	OpenGWAS database	160,589	1388	159,201	East Asian
ebi-a-GCST90018841	OpenGWAS database	476,306	998	475,308	European
finngen_R5_C3_OESOPHAGUS	FinnGen database	218,792	232	218,560	European

EC = esophageal cancer, GWAS = Genome-Wide Association Study.

## 5. IV selection

According to recent studies, in order to enhance the reliability of IVs and ensure the stability of study data and the accuracy of results, IVs need to meet the following criteria: First, SNPs weakly correlated or even uncorrelated with exposure should be excluded. In the forward MR analysis, we used a less stringent threshold of *P* < 1 × 10⁻^5^ for SNP selection. Although a more stringent threshold of *P* < 5e-8 is commonly used in genetic studies, it would have resulted in too few SNPs for effective analysis. Therefore, following recent studies, we used a less stringent threshold of *P* < 1e-5 to ensure sufficient SNPs for robust instrument selection, while research has shown that this threshold still maintains data stability and accuracy.^[24,27–30]^ For the reverse MR analysis, we applied the more stringent threshold of *P* < 5e-8, which is commonly used in genetic studies. This was done to ensure a high level of reliability and minimize the risk of false-positive findings.

Second, To eliminate linkage disequilibrium within IVs and meet the requirements of MR analysis, IVs must satisfy the condition of *R*^2^ < 0.001 and linkage disequilibrium = 10,000 kb.^[31]^ Third, to ensure a robust association of selected IVs with exposure, the strength of genetic variation as IVs should be assessed using the *F* statistic (screening criterion: *F* > 10).^[32]^ The *F* statistic was calculated using the formula


$$\text{F}=\frac{\left(\text{N}-\text{K}-1\right)\cdot {\text{ R}}^{2}}{\text{K}\cdot \;\left(1-{\text{R}}^{2}\right)},$$

where *N* is the sample size in the exposure database, *K* is the number of IVs, and *R*^2^ is the proportion of variance explained by SNPs in the exposure database. The formula for calculating *R*^2^ is


$${\text{R}}^{2}=\frac{\beta^{2}\cdot \text{EAF}\cdot \left(1-\text{EAF}\right)}{{\text{SE}}^{2}\cdot N},$$

where EAF is the effect allele frequency, *β* is the allele effect value, *N* is the sample size, and SE is the standard error. Finally, when combining the exposure and outcome datasets, it is essential to eliminate incompatible alleles and palindromic SNPs.^[33]^

## 6. Power analysis

To ensure the robustness of our findings, we conducted a power analysis using the mRnd tool (http://glimmer.rstudio.com/kn3in/mRnd/).^[34]^ We selected a threshold of ≥80% power, which is widely accepted in genetic epidemiology studies. This ensures that our study has sufficient power to detect true causal effects between immune cell phenotypes, circulating inflammatory proteins, and EC.

## 7. Statistical analysis

### 7.1. MR and BWMR analyses

We conducted a 2-sample MR analysis to investigate causal relationships between 731 immune cell phenotypes, 91 circulating inflammatory proteins, and EC. The primary analytical method was inverse variance weighting (IVW).^[35]^ Additionally, we employed 4 other methods: simple mode,^[36]^ weighted median,^[37]^ weighted mode, and MR-Egger regression,^[38]^ to assess the robustness of the results. BWMR analysis addresses polygenic structure and pleiotropy, providing a complementary approach to traditional MR.^[39]^ There were no overlapping individuals between the exposure (European ancestry) and outcome (Asian ancestry) samples. The distinct genetic backgrounds and lack of individual overlap eliminate potential bias, ensuring the independence of samples and validity of the MR analysis.

## 8. Independent validation

We validated positive MR results in the European cohort and FinnGen database using the IVW method.

## 9. Mediation analysis

We performed a mediation analysis using 2-step MR to explore whether circulating inflammatory proteins mediate the association between immune cells and EC. We calculated the total effect of immune cells on EC, the effect of immune cells on inflammatory proteins (*β*1), and the effect of inflammatory proteins on EC (*β*2). The mediating effect was *β*1*β2, and the direct effect was the total effect minus the mediating effect.^[40]^ By dividing the indirect effect by the total effect, we determined the percentage mediated. The 95% confidence interval (CI) was calculated using the delta method.

## 10. Sensitivity analysis

Heterogeneity among genetic variation estimates was assessed using Cochran *Q* test, with no significant heterogeneity observed at *P* > .05.^[41]^ The MR-Egger intercept assessed horizontal pleiotropy; a *P* value >.05 indicated no pleiotropy.^[42]^ The MR-PRESSO method was used to evaluate and correct pleiotropy, conducted with the R package “MRPRESSO.”^[43]^ Sensitivity analysis used a “leave-one-out” approach to assess the influence of individual IVs on MR results. Forest plots, scatter plots, and funnel plots were generated to visualize sensitivity analysis.

All analyses and data visualization were performed using R (version 4.3.2). Univariable MR analysis was conducted with the R packages “TwoSampleMR” and “MendelianRandomization.”

## 11. Results

In this study, we selected 18,621 SNPs associated with 731 immune cell traits and 2999 SNPs associated with circulating inflammatory proteins to serve as IVs. The detailed characteristics of these SNPs are presented in S3 Table, Supplemental Digital Content, http://links.lww.com/MD/N854 and S4 Table, Supplemental Digital Content, http://links.lww.com/MD/N855. For the reverse MR analysis, we selected 7 SNPs as IVs. Detailed information on these SNPs is provided in S5 Table, Supplemental Digital Content, http://links.lww.com/MD/N856. We conducted a power analysis using the mRnd tool. The results showed that all analyses had ≥80% power to detect causal effects. This ensures that our findings are robust and not influenced by statistical limitations.

## 12. Causal effect of immune cell phenotypes on EC

**Figure 2.**
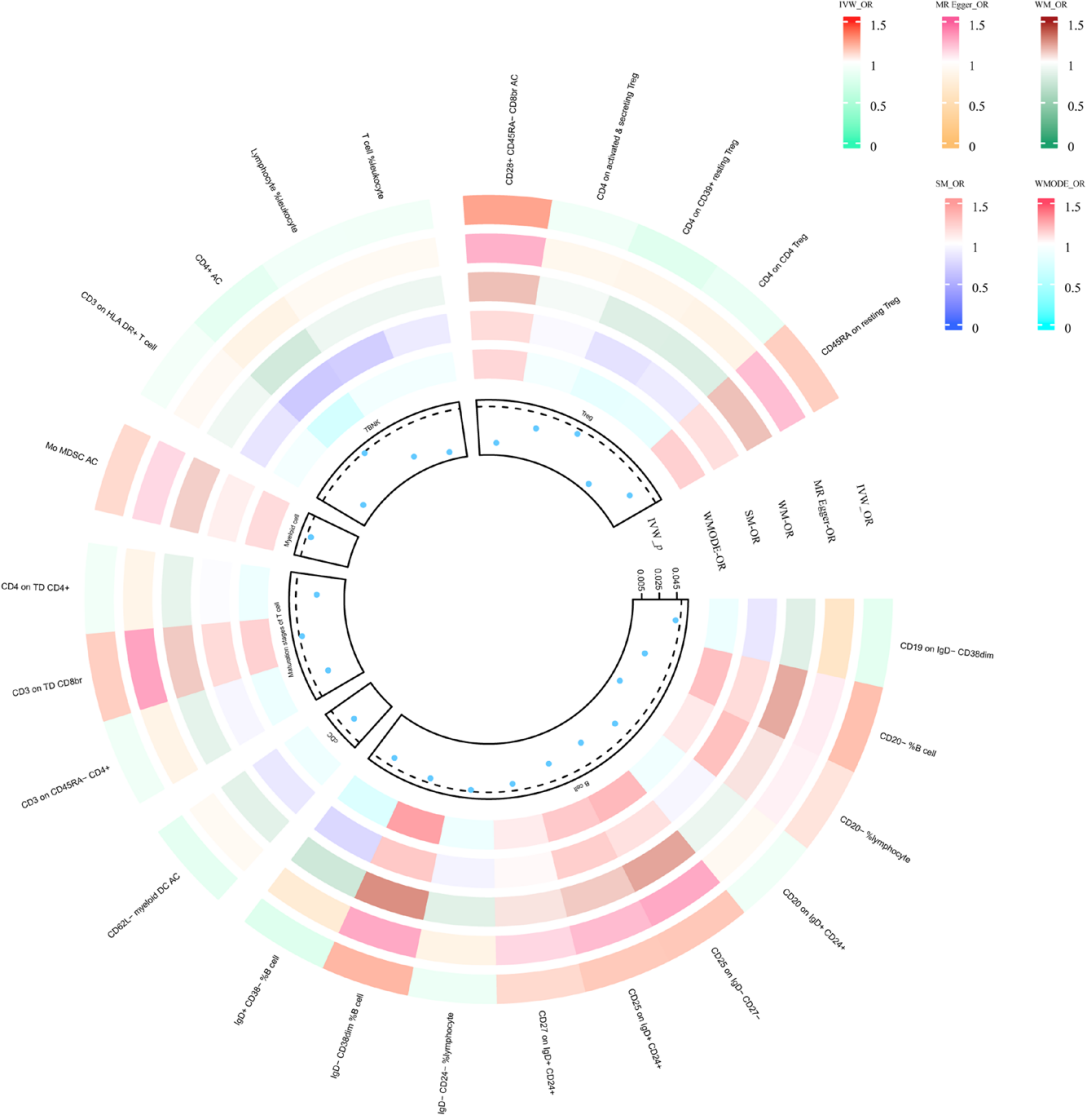
Circle heat map showing significant causal relationships between 24 immune cell traits and EC. EC = East Asian cohorts, IVW = inverse variance weighting, OR = odds ratio, SM = simple mode, WM = weighted median, WMODE = weighted mode.

**Figure 3.**
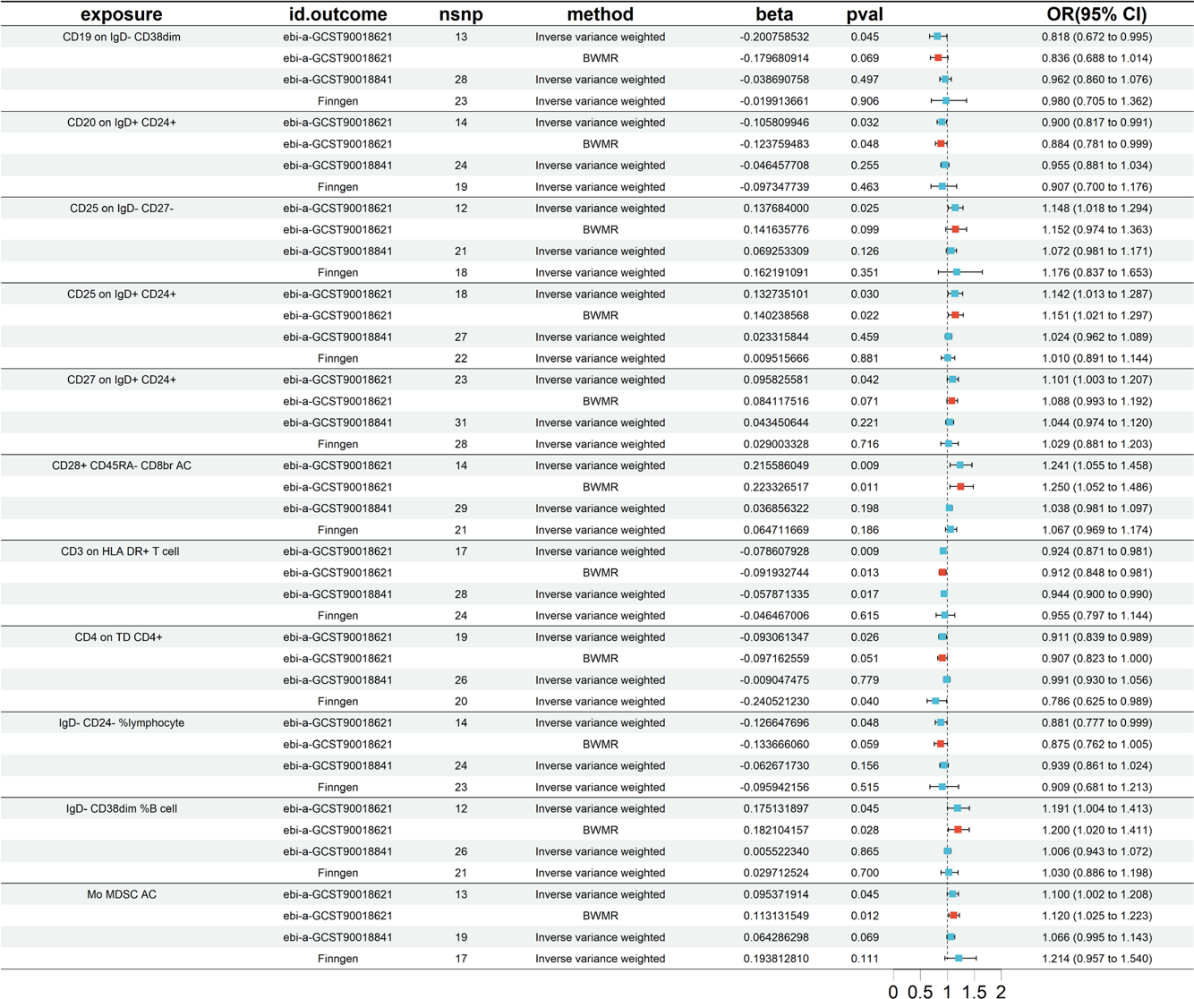
Forest plots showing causal relationships between 11 immune cell traits and esophageal cancer (independent validation). BWMR = Bayesian-Weighted Mendelian Randomization, CI = confidence interval, nsnp = number of single-nucleotide polymorphism, OR = odds ratio.

Our MR analysis identified significant causal relationships between 24 immune cell traits and EC, as shown in Figure 2. These findings were validated in the European cohort and FinnGen database using IVW method. From this rigorous process, we identified 11 immune cell phenotypes of interest: CD19 on IgD- CD38dim (odds ratio [OR] = 0.818, 95% CI = 0.672–0.995, *P* = .045); CD20 on IgD+ CD24+ (OR = 0.900, 95% CI = 0.817–0.991, *P* = .032); CD25 on IgD- CD27- (OR = 1.1476, 95% CI = 1.0178–1.294, *P* = .0246); CD25 on IgD+ CD24+ (OR = 1.142, 95% CI = 1.013–1.287, *P* = .030); CD27 on IgD+ CD24+ (OR = 1.101, 95% CI = 1.003–1.207, *P* = .042); CD28+ CD45RA− CD8br absolute count (AC) (OR = 1.241, 95% CI = 1.055–1.458, *P* = .009); CD3 on human leukocyte antigen (HLA) DR+ T cell (OR = 0.924, 95% CI = 0.871–0.981, *P* = .009); CD4 on TD CD4+ (OR = 0.911, 95% CI = 0.839–0.989, *P* = .026); 9) IgD- CD24- %lymphocyte (OR = 0.881, 95% CI = 0.777–0.999, *P* = .0482); IgD- CD38dim %B cell (OR = 1.191, 95% CI = 1.004–1.413, *P* = .045); monocytic myeloid-derived suppressor cells (Mo-MDSC) AC (OR = 1.100, 95% CI = 1.00–1.208, *P* = .045). Figure 3 illustrates these results. Reverse MR analysis showed no significant causal relationship between EC and the 11 immune cell traits identified. Our findings were further supported by scatter plots (S1 Fig, Supplemental Digital Content, http://links.lww.com/MD/N857), leave-one-out analysis (S2 Fig, Supplemental Digital Content, http://links.lww.com/MD/N857), and funnel plots (S3 Fig, Supplemental Digital Content, http://links.lww.com/MD/N857). No significant heterogeneity or horizontal pleiotropy was detected (*P* > .05), as detailed in Table 2, confirming the reliability of our results.

**Table 2:** Sensitivity analysis of causal relationship between 11 immune cell traits and EC.

Exposure	Outcome	Method	Cochran *Q*			Pleiotropy		
			*Q*	*Q*_df	*Q*_pval	Egger intercept	SE	pval
CD19 on IgD- CD38dim	EC	MR Egger	7.03	11	0.80	0.0433	0.0567	0.46
		IVW	7.61	12	0.81			
		MR-PRESSO						0.79
CD20 on IgD+ CD24+	EC	MR Egger	11.84	12	0.46	−0.0038	0.0223	0.87
		IVW	11.87	13	0.54			
		MR-PRESSO						0.70
CD25 on IgD- CD27-	EC	MR Egger	14.86	10	0.14	−0.0174	0.0384	0.66
		IVW	15.17	11	0.18			
		MR-PRESSO						0.28
CD25 on IgD+ CD24+	EC	MR Egger	8.07	11	0.71	−0.0070	0.0321	0.83
		IVW	8.12	12	0.78			
		MR-PRESSO						0.80
CD27 on IgD+ CD24+	EC	MR Egger	10.14	12	0.60	−0.0017	0.0467	0.97
		IVW	10.14	13	0.68			
		MR-PRESSO						0.69
CD28+ CD45RA- CD8br AC	EC	MR Egger	19.94	16	0.22	−0.0195	0.0319	0.55
		IVW	20.40	17	0.25			
		MR-PRESSO						0.27
CD3 on HLA DR+ T cell	EC	MR Egger	11.27	15	0.73	0.0049	0.0177	0.79
		IVW	11.35	16	0.79			
		MR-PRESSO						0.87
CD4 on TD CD4+	EC	MR Egger	13.69	17	0.69	0.0251	0.0186	0.20
		IVW	15.51	18	0.63			
		MR-PRESSO						0.64
IgD- CD24- %lymphocyte	EC	MR Egger	8.14	12	0.77	0.0150	0.0273	0.59
		IVW	8.44	13	0.81			
		MR-PRESSO						0.82
IgD- CD38dim %B cell	EC	MR Egger	25.49	21	0.23	−0.0089	0.0219	0.69
		IVW	25.69	22	0.27			
		MR-PRESSO						0.26
Mo-MDSC AC	EC	MR Egger	4.32	12	0.98	−0.0389	0.0329	0.26
		IVW	5.72	13	0.96			
		MR-PRESSO						0.96

EC = esophageal cancer, IVW = inverse variance weighted, MR-PRESSO = MR-Pleiotropy Residual Sum and Outlier, SE = standard error.

## 13. Causal associations between circulating inflammatory proteins and EC

**Figure 4.**
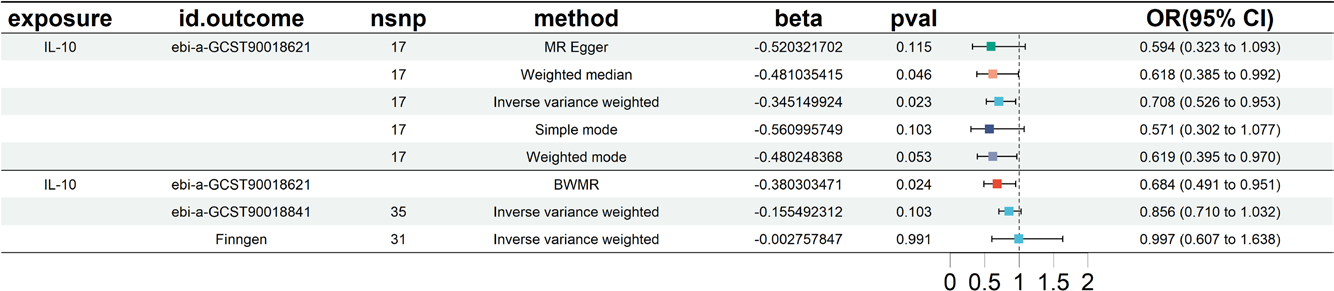
Forest plots showing causal relationships between IL-10 and esophageal cancer. BWMR = Bayesian-Weighted Mendelian Randomization, CI = confidence interval, IL = interleukin, nsnp = number of single-nucleotide polymorphism, OR = odds ratio.

Our tests indicated no significant heterogeneity or horizontal pleiotropy among the IVs (*P* > .05), as shown in Table 3. Therefore, we used the outcomes of the IVW analysis as the results of the MR analysis, employing a fixed-effects model for the final findings. The MR analysis, combined with BWMR analysis, revealed a negative correlation between interleukin-10 (IL-10) levels and EC risk (IVW OR = 1.117, 95% CI = 1.008–1.237, *P* = .035). These findings were validated in the European cohort and the FinnGen database, yielding consistent results, as shown in Figure 4. Reverse MR analysis found no significant causal relationship between EC and IL-10 levels. The supporting scatter plots, leave-one-out test, and funnel plots are available in S4 Fig, Supplemental Digital Content, http://links.lww.com/MD/N857.

**Table 3:** Sensitivity analysis of causal relationship between IL-10 and EC.

Exposure	Outcome	Method	Cochran *Q*			Pleiotropy		
			*Q*	*Q*_df	Q_pval	Egger intercept	SE	pval
IL-10	EC	MR Egger	12.18	15	0.67	0.0172	0.0266	0.53
		IVW	12.60	16	0.70			
		MR-PRESSO						0.55

EC = esophageal cancer, IL = interleukin, IVW = inverse variance weighted, MR-PRESSO = MR-Pleiotropy Residual Sum and Outlier, SE = standard error.

## 14. Causal effect of immune cell phenotypes on IL-10

**Figure 5.**
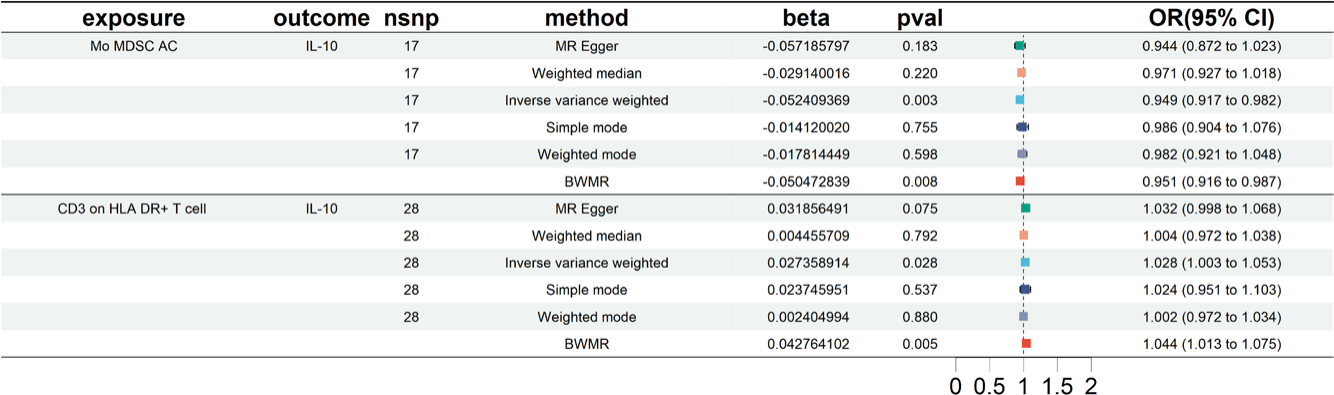
Forest plots showing causal relationships between 2 immune cell traits and IL-10. BWMR = Bayesian-Weighted Mendelian Randomization, CI = confidence interval, IL = interleukin, Mo-MDSC = monocytic myeloid-derived suppressor cell, nsnp = number of single-nucleotide polymorphism, OR = odds ratio.

The study identified significant associations between 2 immune cell phenotypes and IL-10 levels. As shown in Figure 5, the following causal relationship was observed: Mo-MDSC AC exhibited a negative correlation with IL-10 levels (OR = 0.949, 95% CI = 0.917–0.982, *P* = .0028). CD3 on HLA DR+ T cells showed a positive correlation with IL-10 levels (OR = 1.028, 95% CI = 1.003–1.053, *P* = .0279). No significant heterogeneity or horizontal pleiotropy was detected (*P* > .05), as detailed in Table 4. Supporting visualizations, including scatter plots, leave-one-out tests, and funnel plots, are provided in S5 Fig, Supplemental Digital Content, http://links.lww.com/MD/N857.

**Table 4:** Sensitivity analysis of causal relationship between 2 immune cell traits and IL-10.

Exposure	Outcome	Method	Cochran *Q*			Pleiotropy		
			*Q*	*Q*_df	*Q*_pval	Egger intercept	SE	pval
Mo-MDSC AC	IL-10	MR Egger	23.50	15	0.07	0.0015	0.0112	0.90
		IVW	23.52	16	0.10			
		MR-PRESSO						0.08
CD3 on HLA DR+ T cell	IL-10	MR Egger	37.69	26	0.06	−0.0022	0.0058	0.70
		IVW	37.91	27	0.08			
		MR-PRESSO						0.07

HLA-DR = human leukocyte antigen-DR, IL = interleukin, IVW = inverse variance weighted, Mo-MDSC = monocytic myeloid-derived suppressor cell, MR-PRESSO = MR-Pleiotropy Residual Sum and Outlier.

## 15. Mediation analysis

In our final analysis, we performed a mediation analysis to explore the causal relationship between immune cell phenotypes and EC, with IL-10 levels serving as a mediator. We found that IL-10 levels mediated the relationship between 2 immune cell phenotypes and EC.

**Figure 6.**
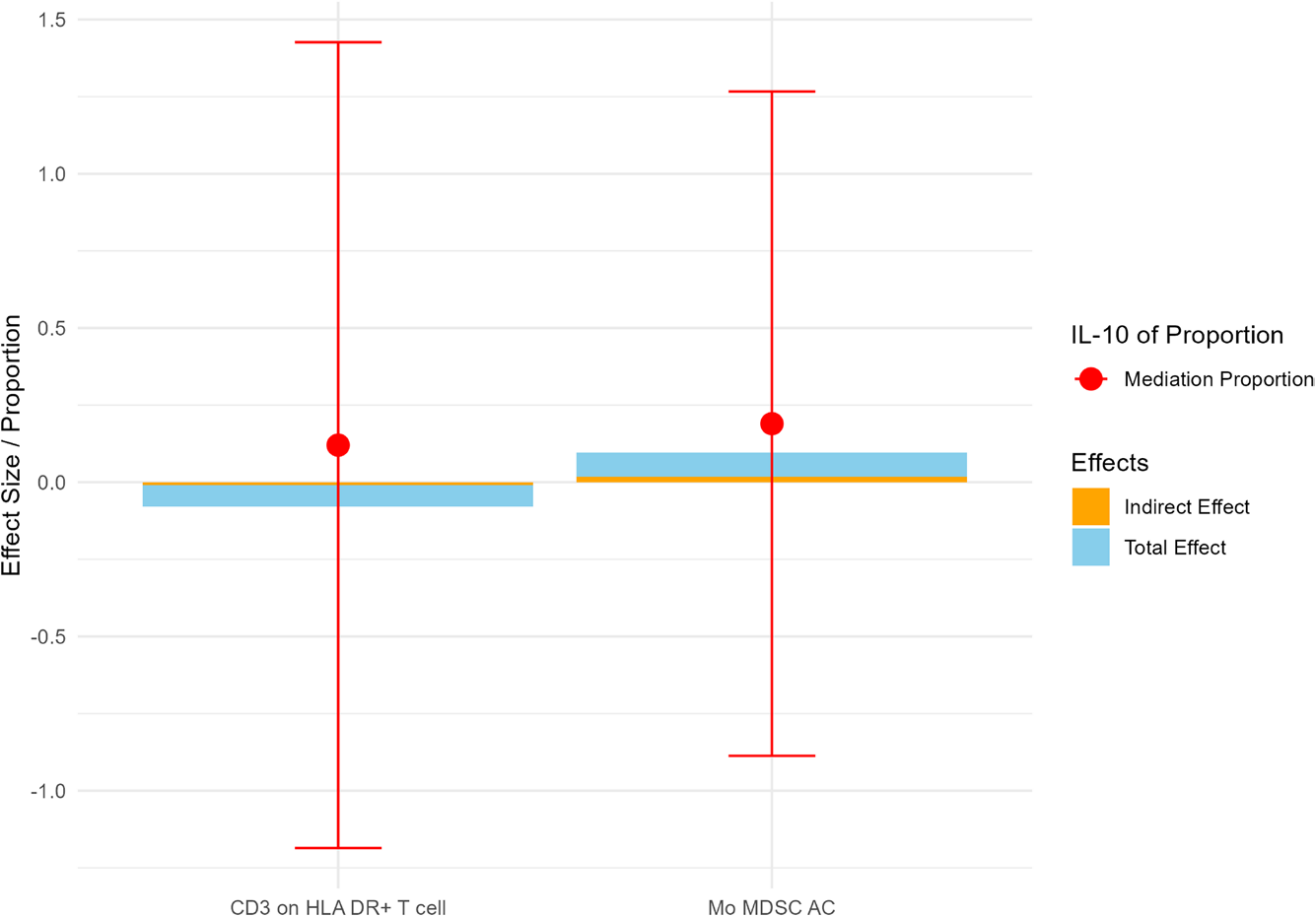
Mediating effects of IL-10 on the causal relationship between 2 immune cell phenotypes and esophageal cancer. HLA-DR = human leukocyte antigen-DR, IL = interleukin, Mo-MDSC = monocytic myeloid-derived suppressor cell.

Specifically, the mediation effect of IL-10 levels in the causal pathway from Mo-MDSC AC to EC was 0.018 (95% CI = −0.085 to 0.121), accounting for 18.97% of the total effect. Similarly, the mediation effect of IL-10 levels from CD3 on HLA DR+ T cells to EC was −0.009 (95% CI = −0.112 to 0.093), accounting for 12.01% of the total effect (Fig. 6, Table 5).

**Table 5:** Mediating effects of IL-10 on the causal relationship between 2 immune cell phenotypes and esophageal cancer.

Exposure	Total effect	Direct effect A	Direct effect B	Indirect effect	Mediation proportion (%)
		*β*1	*β*2	*β*1*β2	(95% CI)
CD3 on HLA DR+ T cell	−0.0786	0.0274	−0.3451	−0.0094	12.01 (1.43 to −1.19)
Mo-MDSC AC	0.0954	−0.0472	−0.3451	0.0163	17.10 (−0.91 to 1.25)

Total effect, Causal effect of immune cell phenotypes on esophageal cancer; Direct effect A, Causal effect of immune cell phenotype on circulating inflammatory proteins; Direct effect B, Causal effect of circulating inflammatory proteins on esophageal cancer; Indirect effect = direct effect A*direct effect B; Mediation proportion = mediate effect/total effect.

CI = confidence interval, HLA-DR = human leukocyte antigen-DR, IL = interleukin, Mo-MDSC = monocytic myeloid-derived suppressor cell.

## 16. Discussion

In this study, we conducted multiple 2-sample MR analyses to explore the causal effects of 731 immune cell phenotypes and 91 circulating inflammatory proteins on EC risk. We identified significant causal relationships for 11 immune cell phenotypes and one inflammatory cytokine, IL-10, with EC. These immune cell phenotypes include CD19 on IgD- CD38dim, CD20 on IgD +CD24+, CD25 on IgD- CD27-, CD25 on IgD+ CD24+, CD27 on IgD+ CD24+, CD28+ CD45RA− CD8br AC, CD3 on HLA DR+ T cells, CD4 on TD CD4+, IgD- CD24- %lymphocytes, IgD- CD38dim %B cells, and Mo-MDSC AC. However, further mediation analyses revealed that only 2 cell phenotypes, CD3 on HLA DR+ T cells and Mo-MDSC AC, were causally linked to EC through IL-10. Specifically, CD3 on HLA DR+ T cells had a mediation effect of −0.009 (mediation proportion: 12.01%), while Mo-MDSC AC had a mediation effect of 0.018 (mediation proportion: 18.97%).

IL-10, located on chromosome 1q32.2,^[44]^ is a crucial anti-inflammatory cytokine that plays a significant role in modulating immune responses.^[45]^ It reduces chronic inflammation by inhibiting the production of pro-inflammatory cytokines such as tumor necrosis factor-α, IL-1, IL-6, and IL-12.^[46]^ Chronic inflammation, characterized by persistent tissue damage and repair, significantly increases the risk of EC.^[47]^ During chronic inflammation, inflammatory mediators like tumor necrosis factor-α and IL-6,^[48]^ along with signaling molecules such as nuclear factor-kappa B,^[49]^ are activated. These factors promote cell proliferation and inhibit apoptosis, enhancing cancer cell survival and increasing the risk of malignancy. Furthermore, chronic inflammation creates an environment that allows tumor cells to evade immune surveillance, facilitating their growth and survival.^[50]^ IL-10’s anti-inflammatory properties may counteract these processes, highlighting its potential protective role.

Our study found a significant inverse correlation between IL-10 levels and the risk of EC, suggesting that IL-10 may protect against EC by reducing chronic inflammation. However, other studies have shown that IL-10 levels are elevated in EC patients and are associated with decreased survival rates.^[51]^ Despite this, our survival analysis indicated that IL-10 levels were not an independent risk factor for reduced survival rates. Additionally, research indicates that while IL-10 levels increase in EC patients, co-administration of IL-10 with crizotinib effectively reduces PD-L1 expression in EC.^[52]^ Our study focused on evaluating the risk of EC before its onset. We propose that IL-10 may play different roles at various stages of cancer development. Prior to the onset of EC, IL-10 likely acts as a protective factor by reducing chronic inflammation and, consequently, lowering the risk of cancer development. However, after EC has developed, elevated IL-10 levels might contribute to more complex interactions within the tumor microenvironment. These increased IL-10 levels could potentially support tumor progression by suppressing effective immune responses against cancer cells. Therefore, further research is necessary to fully understand the dual roles of IL-10 in both the prevention and progression of EC.

CD3 on HLA-DR+ T cells indicates an activated subset of T cells. HLA-DR is an major histocompatibility complex class II molecule encoded by the HLA complex in the 6p21 region.^[53,54]^ It is typically expressed by antigen-presenting cells, including DCs, macrophages, and B cells.^[55]^ HLA-DR plays a key role in presenting antigens to CD4+ T cells and activating them, which in turn trigger immune responses against tumors.^[56]^ Studies in colorectal cancer have identified HLA-DR as an important prognostic marker of good outcomes.^[57]^ In esophageal adenocarcinoma, patients with high HLA-DR expression at the tumor core and invasive front have significantly better survival outcomes compared to those with low HLA-DR expression in tumor epithelium.^[58]^ Additionally, HLA-DR levels have been noted to increase substantially following immunotherapy in EC patients.^[59]^

Our study found that CD3 on HLA-DR+ T cells was negatively correlated with the risk of EC, suggesting that an increase in these cells may help reduce the risk of developing EC, consistent with previous research. Mediation analysis indicated that IL-10 might be a potential mediator in the relationship between CD3 on HLA-DR +T cells and EC risk. Interestingly, CD3 on HLA-DR+ T cells is positively correlated with IL-10 levels. We hypothesize that CD3 on HLA-DR+ T cells promotes inflammatory responses in anti-tumor immunity through cytokine secretion. IL-10 secretion may trigger a feed-forward signaling mechanism that fosters the development of self-limiting adaptive IL-10-producing T cells.^[45,60]^ These self-limiting T cells produce IL-10 at appropriate times to suppress excessive immune responses, preventing potential damage to the host. Consequently, in anti-tumor immunity, CD3 on HLA-DR+ T cells not only enhances inflammatory responses but also helps maintain immune homeostasis via the IL-10 feed-forward regulatory mechanism. This dual role protects the host from the adverse effects of overly robust immune responses.

Mo-MDSCs are a specific type of immunoregulatory cell classified as a subset of myeloid cells.^[61]^ Their primary characteristic is their immunosuppressive function, which inhibits the activity of other immune cells through various mechanisms. Mo-MDSCs produce large amounts of nitric oxide,^[62]^ arginase I,^[63]^ and immunosuppressive cytokines, including IL-10 and transforming growth factor-β.^[64]^ These substances suppress both antigen-specific and nonspecific T-cell responses, thereby regulating and inhibiting immune reactions.^[65]^ As a result, they promote a tolerogenic microenvironment, protecting the host from uncontrolled inflammation. During cancer, MDSCs rapidly expand, suppressing both innate and adaptive immunity and playing a crucial role in tumor immune evasion.^[66]^ Studies show that the quantity of MDSCs in peripheral blood correlates positively with cancer stage and tumor burden.^[67-69]^ Mechanistically, active arginase I expressed by MDSCs inhibits CD8 T cells from secreting interferon-γ and IL-2, thereby promoting tumor growth and progression.^[70]^ Additionally, MDSCs induce epithelial-mesenchymal transition in cancer cells through signaling pathways like transforming growth factor-β and epidermal growth factor, promoting cancer cell dissemination.^[71]^ Targeting the immunosuppressive mechanisms of MDSCs could potentially enhance the response rates of immune checkpoint inhibitor therapy.^[72]^ Our study identified a positive correlation between Mo-MDSC AC and EC. Increased Mo-MDSC AC levels may elevate the risk of EC by suppressing anti-tumor immune responses, consistent with previous studies. Mediation analysis suggested IL-10 as a potential mediator in the effect of Mo-MDSC AC on EC risk. Interestingly, our study found a negative correlation between Mo-MDSC AC and IL-10 levels, possibly due to a negative feedback regulation mechanism. Although Mo-MDSC AC secretes IL-10, IL-10 might inhibit the function of Mo-MDSC AC itself or nearby Mo-MDSC ACs through its receptor (IL-10R) via autocrine and paracrine pathways.^[73]^ After binding to its receptor, IL-10 inhibits the activation state of Mo-MDSC AC and further IL-10 secretion by activating downstream signaling pathways such as STAT3.^[74]^ Additionally, this negative feedback mechanism might lead to IL-10 inhibiting the secretion of IL-10 by other immune cells such as macrophages, DCs, and T cells, thereby reducing overall IL-10 levels. While this helps avoid excessive immunosuppression and maintains immune system balance, it could also increase the risk of chronic inflammation and EC.

Our study has several strengths. First, we investigated the causal relationships between 731 immune cell phenotypes, 91 circulating inflammatory proteins, and EC, providing a comprehensive overview of the potential interactions. Our mediation analyses revealed that 2 immune cell phenotypes could be causally linked to EC through IL-10. Additionally, we found that IL-10 was negatively associated with esophageal tumorigenesis, suggesting its potential protective role against cancer development. These findings are intriguing and warrant further investigation. However, it is important to acknowledge the study’s limitations. We cannot entirely rule out the influence of confounding factors that may affect our results. Validating our findings across different ethnic groups could enhance the general applicability of our conclusions but may also introduce population stratification bias. The lack of individual-specific data prevented us from conducting a more detailed stratified analysis, which may have led to inaccuracies in our conclusions. Moreover, the interactions between immune cells and inflammatory proteins are inherently complex and involve both positive and negative feedback mechanisms. This complexity necessitates further basic and clinical research to fully understand their roles in EC.

## 17. Conclusions

In conclusion, our comprehensive 2-sample MR and mediation Mendelian analyses demonstrated that the causal relationship between 2 immunophenotypes (CD3 on HLA DR+ T cells and Mo-MDSC AC) and EC is mediated through IL-10, highlighting the complex interactions between the immune system and EC. Additionally, we found that IL-10 might play a crucial role in reducing esophageal carcinogenesis. Our findings provided valuable insights for immunotherapy studies in EC. This might provide researchers with valuable insights for further exploration of immunotherapy in EC. We looked forward to subsequent experimental verification emphasizing the causal role of immune cell phenotypes and IL-10 in EC risk.

## Acknowledgments

Thanks to all authors for their contributions.

## Author contributions

**Conceptualization:** Jinzhou Guo, Fuchun Si.

**Data curation:** Jinzhou Guo.

**Investigation:** Jinzhou Guo, Gao Si, Xuejie Song.

**Methodology:** Jinzhou Guo, Fuchun Si.

**Software:** Jinzhou Guo, Gao Si.

**Visualization:** Jinzhou Guo, Gao Si.

**Writing – original draft:** Jinzhou Guo.

**Formal analysis:** Jinzhou Guo, Gao Si.

**Validation:** Gao Si, Xuejie Song.

**Supervision:** Xuejie Song, Fuchun Si.

**Writing – review & editing:** Xuejie Song, Fuchun Si.

## Supplementary Material


