# Supplementary material for: Mediating role of circulating inflammatory proteins in the effect of immune cells on esophageal cancer risk: A Mendelian randomization study
Source: Medicine (Baltimore). 2024 Nov 1;103(44):e40374. doi: 10.1097/MD.0000000000040374 (PMC11537666; doi:10.1097/MD.0000000000040374)
Supplement: Supplementary file 6 [file medi-103-e40374-s006.docx]

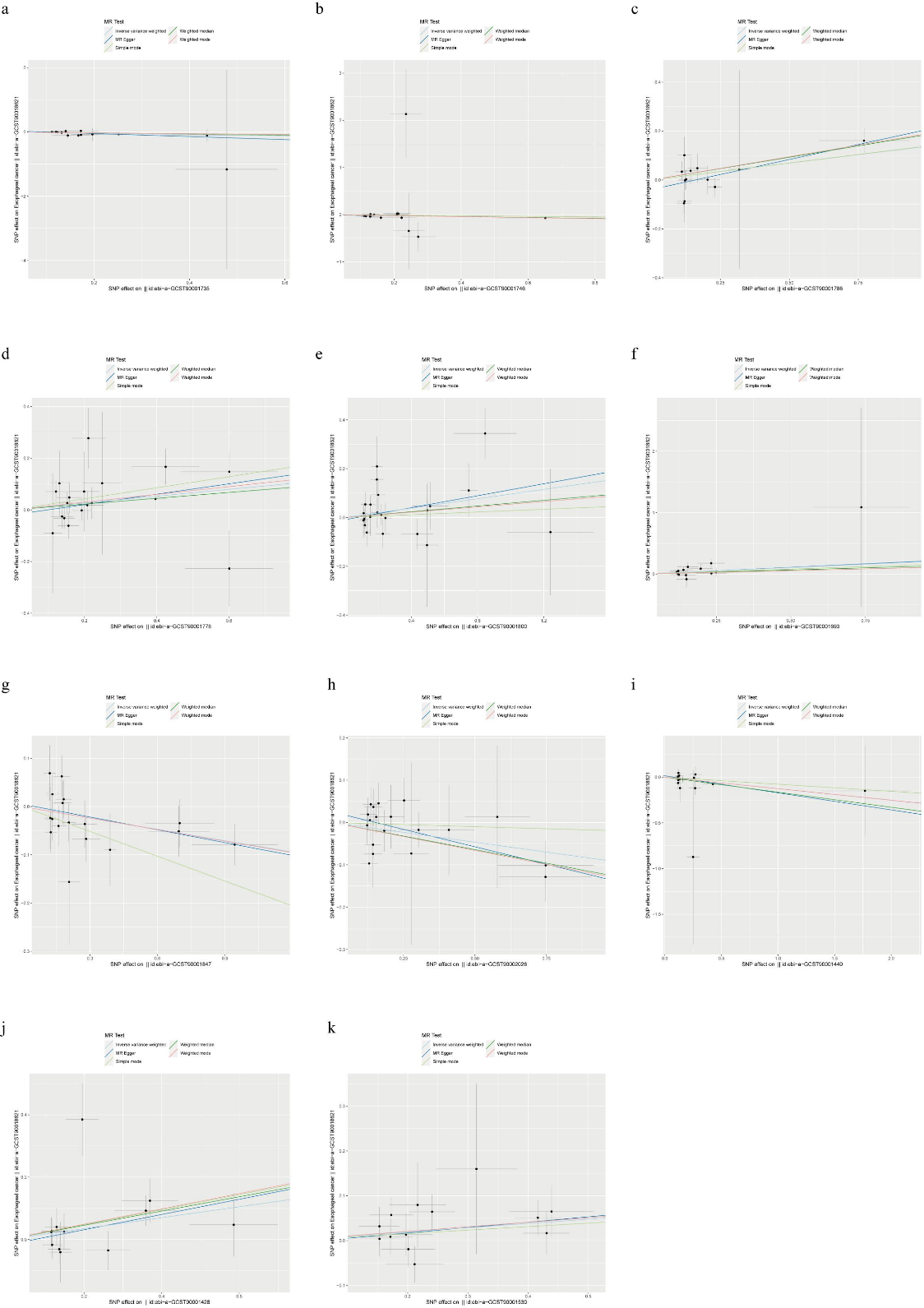


**S1 Fig. Scatter plots of Associations Between 11 Immune Cell Traits and EC.** (a) CD19 on IgD- CD38dim on EC; (b) CD20 on IgD+ CD24+ on EC; (c) CD25 on IgD- CD27- on EC; (d) CD25 on IgD+ CD24+ on EC; (e) CD27 on IgD+ CD24+ on EC; (f) CD28+ CD45RA- CD8br ACon EC; (g) CD3 on HLA DR+ T cell on EC; (h) CD4 on TD CD4+ on EC; (i) IgD-CD24- % lymphocyte on EC; (j) IgD- CD38dim %B cell on EC; (k) Mo MDSC AC on EC.


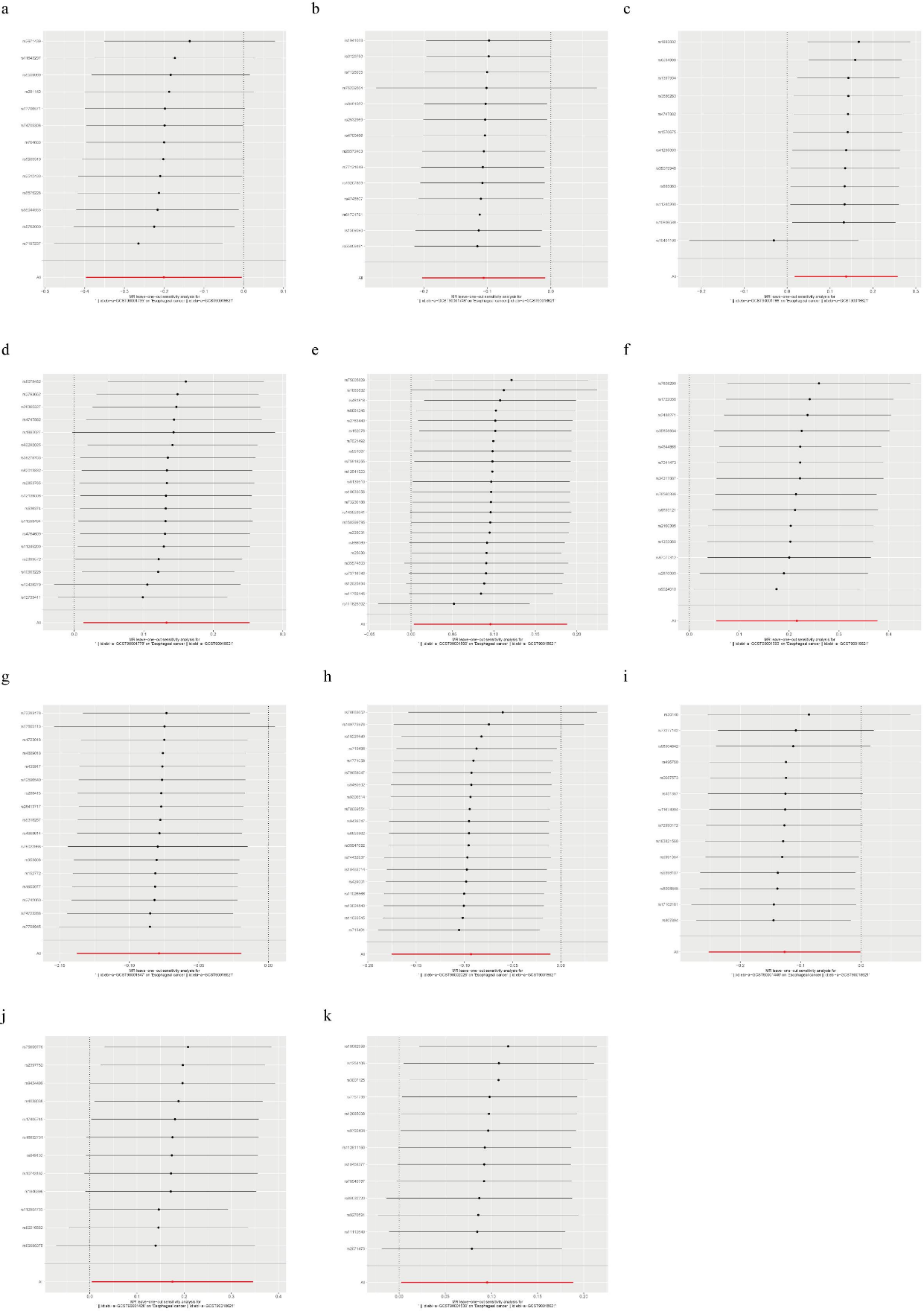


**S2 Fig. Leave-One-Out Sensitivity Analysis of Associations Between 11 Immune Cell Traits and**

**EC.** (a) CD19 on IgD- CD38dim on EC; (b) CD20 on IgD+ CD24+ on EC; (c) CD25on IgD- CD27-

on EC; (d) CD25 on IgD+ CD24+ on EC; (e) CD27 on IgD+ CD24+ on EC; (f) CD28+CD45RA CD8br AC on EC; (g) CD3 on HLA DR+ T cell on EC; (h) CD4 on TD CD4+ on EC; (i) IgD-CD24- % lymphocyte on EC; (j) IgD- CD38dim %B cell on EC; (k) Mo MDSC AC on EC.


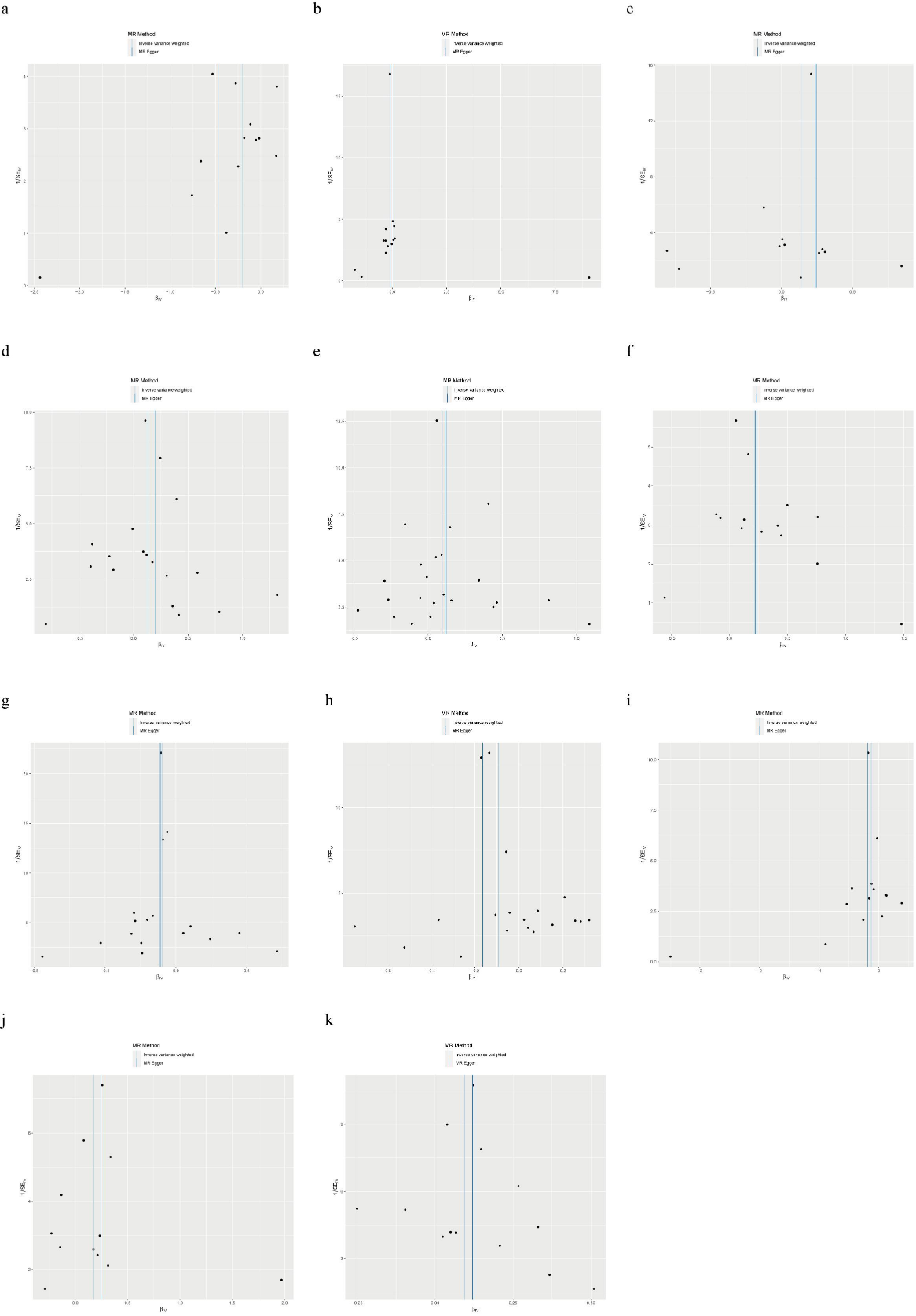


**S3 Fig. Funnel Plots of Associations Between 11 Immune Cell Traits and EC.** (a) CD19 on IgD- CD38dim on EC; (b) CD20 on IgD+ CD24+ on EC; (c) CD25 on IgD- CD27- on EC; (d) CD25 on IgD+ CD24+ on EC; (e) CD27 on IgD+ CD24+ on EC; (f) CD28+ CD45RA- CD8br AC on EC; (g)

CD3 on HLA DR+ T cell on EC; (h) CD4 on TD CD4+ on EC; (i) IgD-CD24- % lymphocyte on EC; (j) IgD- CD38dim %B cell on EC; (k) Mo MDSC AC on EC.


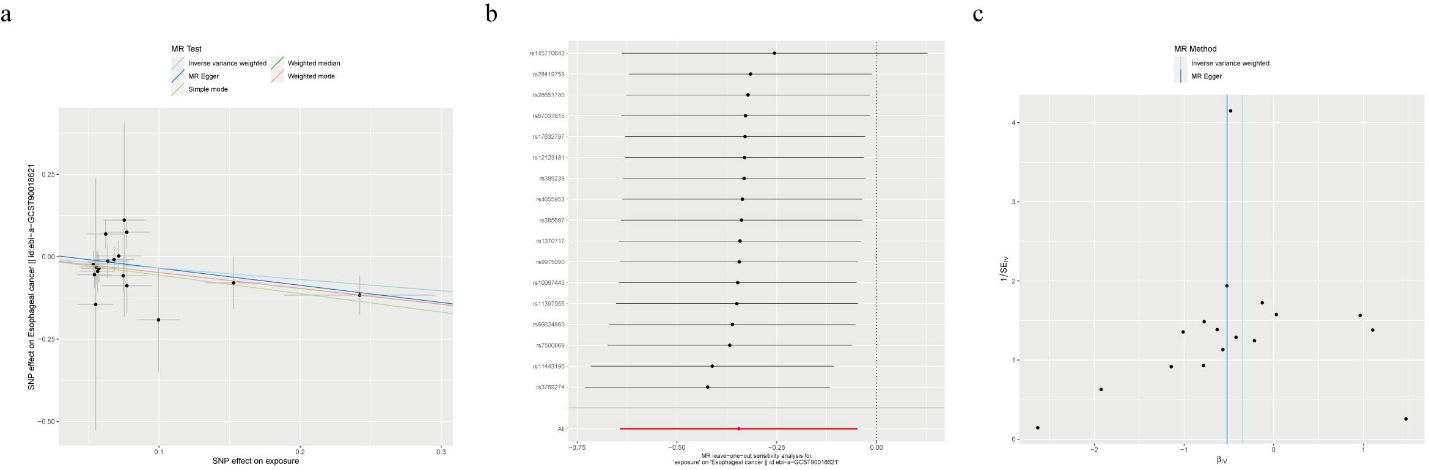


**S4 Fig. Analysis Results of IL-10 and EC Analysis: Scatter Plots, Leave-One-OutTest, and Funnel Plots.** (a) Scatter Plots; (b) Leave-One-Out Test; (c) Funnel Plots.


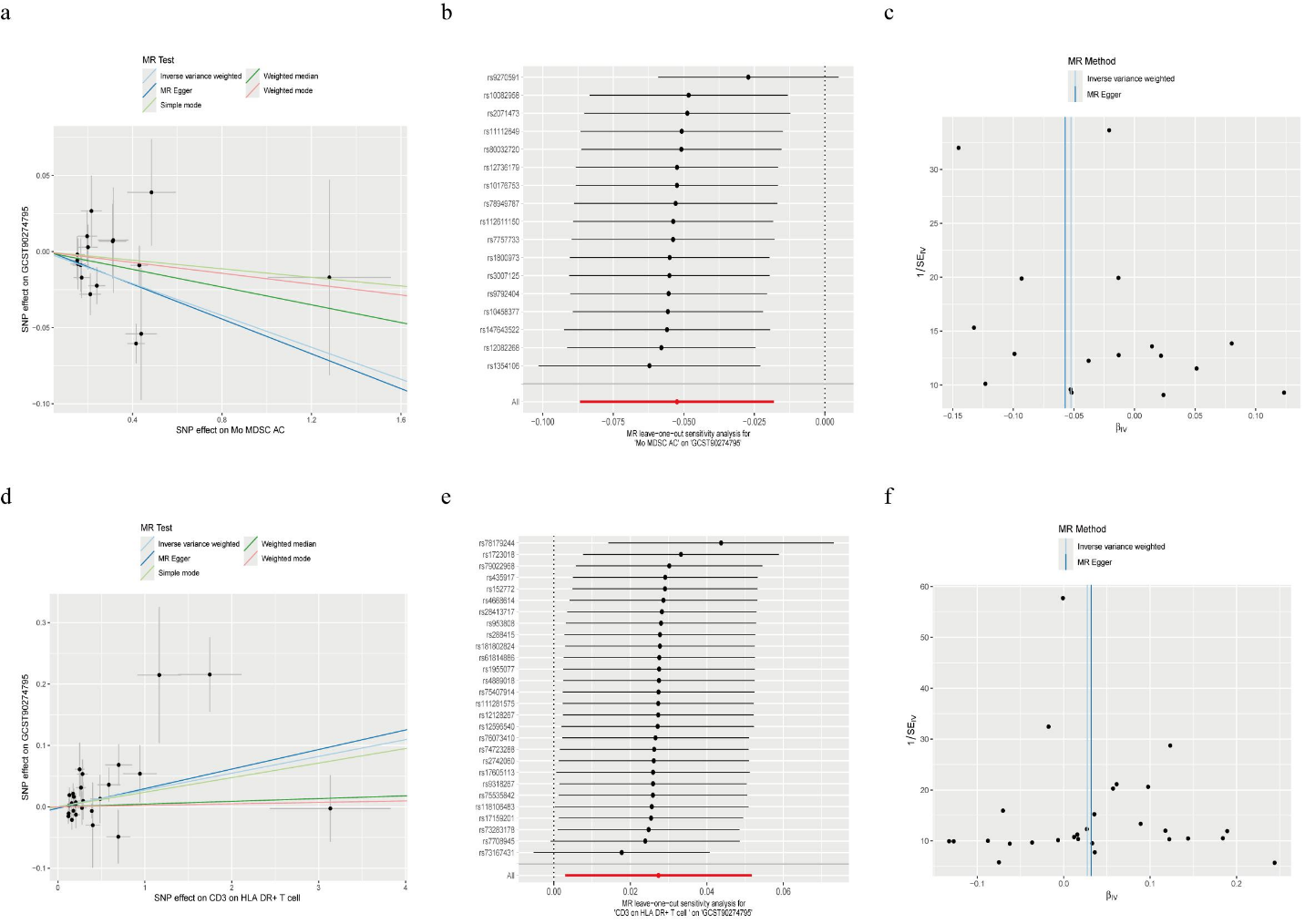


**S5 Fig. Analysis Results of Two Immune Cells and IL-10:Scatter Plots, Leave-One-OutTests, and Funnel Plots.** (a) Scatter Plots of Mo MDSC AC on IL-10; (b) Leave-One-Out Test of Mo MDSC AC on IL-10; (c) Funnel Plots of Mo MDSC AC on IL-10; (d) Scatter Plots of CD3 on HLA DR+ T cell on IL-10; (e) Leave-One-Out Test of CD3 on HLA DR+ T cell on IL-10; (f) Funnel Plots of CD3 on HLA DR+ T cell on IL-10.
